# Unraveling the Predictive Value of *HGF*, *RECK*, *CYP1B1*, and *ZEB2* for Cancer Stemness and Immune Microenvironment in Pancreatic Adenocarcinoma

**DOI:** 10.1155/sci/2402325

**Published:** 2026-05-14

**Authors:** Hongxi Chen, Shenglei Song, Chenglong Li, Zhige Yu

**Affiliations:** ^1^ Department of General Surgery, Hunan Provincial People’s Hospital (The First Affiliated Hospital of Hunan Normal University), Changsha, 410005, China, hunnu.edu.cn

**Keywords:** autophagy, cancer stem cell, immunity, molecular docking, pancreatic adenocarcinoma, resveratrol

## Abstract

**Introduction:**

The progression of pancreatic adenocarcinoma (PAAD) is closely linked to autophagy and microRNA (miRNA) regulation. Therefore, constructing miRNA regulatory networks based on PAAD autophagy‐related genes is essential for improving targeted therapy.

**Methods:**

In this study, we downloaded PAAD‐related data from The Cancer Genome Atlas (TCGA) and Genotype‐Tissue Expression (GTEx), PAAD‐related miRNA data from Gene Expression Omnibus (GEO), and mined autophagy from existing literature reports for autophagy‐related genes. Autophagy signature scores were assessed employing single‐sample gene set enrichment analysis (ssGSEA) and screened for autophagy‐associated candidate genes via weighted gene co‐expression network analysis (WGCNA) and differential expression analysis. Bioinformatics tools such as CIBERSORT and ESTIMATE were employed to assess the level of immune infiltration. The Encori database and Cytoscape 3.8.0 tools were used to construct the miRNA regulatory network. The PubChem website and AutoDockTools were used for targeted drug prediction and molecular docking of PAAD autophagy‐related genes. Cell counting kit‐8 (CCK‐8), scratch healing assay, transwell assay, etc. were used to investigate the regulatory effect of biomarkers on the PAAD cells.

**Results:**

WGCNA combined differential expression analysis obtained candidate genes related to PAAD autophagy, which were primarily implicated in the pathways of phagocytosis and miRNAs in cancer. Among them, four characteristic genes (*HGF*, *RECK*, *CYP1B1*, and *ZEB2*) contained in the miRNAs in cancer pathway will be included in the subsequent analyses as biomarkers affecting PAAD autophagy. The expressions of these biomarkers were positively linked to all immune‐relevant indicators (Stromal, Immune, and ESTIMATE scores) of PAAD. The regulatory network of miRNAs and four biomarkers in PAAD was constructed, and it was elucidated that hsa‐miR‐222‐3p and hsa‐miR‐377‐3p had targeting relationship to *RECK* and *CYP1B1*, respectively. Moreover, results showed that *HGF*, *RECK*, and *ZEB2* were significantly positively correlated with cancer stem cell (CSC) scores (*R* > 0.3), and the high expressions of *HGF*, *RECK*, and *ZEB2* genes all led to the significant enrichment of EPITHELIAL_MESENCHYMAL_TRANSITION and TGF_BETA_SIGNALING. Molecular docking revealed that *CYP1B1*, *HGF*, and *RECK* all stably bound resveratrol. *ZEB2* is significantly associated with proliferation, migration, and invasion in the PAAD cells.

**Conclusion:**

The present study elucidated genes associated with autophagy features in PAAD by bioinformatics and constructed corresponding miRNA networks and molecular docking models, and predicted potential target drugs for PAAD, which will guide the development of prognostic therapeutic strategies for PAAD.

## 1. Introduction

Pancreatic adenocarcinoma (PAAD) is a malignancy with poor prognosis and a high mortality rate, and most patients are in the advanced stages of the disease when diagnosed owing to the absence of obvious symptoms in the early diagnosis of PAAD [1, 2]. Although conventional therapies have slowed down disease progression to some extent, the therapeutic effects are limited due to the lack of targeted therapeutic targets [3, 4]. The standard treatment for advanced PAAD at present is systemic chemotherapy, whereby regimens like FOLFIRINOX (5‐fluorouracil, leucovorin, irinotecan, and oxaliplatin) and gemcitabine plus nab‐paclitaxel show better survival than gemcitabine [5, 6]. Despite this, the median overall survival for patients with metastatic disease treated with these regimens is only around 11 months with FOLFIRINOX and 8.5 months with gemcitabine plus nab‐paclitaxel [7]. Immunotherapy, while revolutionary in many solid tumors, has shown limited efficacy in unselected PAAD populations, with immune checkpoint inhibitors achieving objective response rates of only 16.2% in systematic analyses, though promising outcomes have been observed in the rare subset of patients with microsatellite instability‐high or mismatch repair‐deficient tumors [8]. Currently, innovative approaches that combine immunotherapy with chemotherapy or radiotherapy or with other agents are being actively tested to combat the immunosuppressive tumor microenvironment (TME) of PAAD [9, 10]. Thus, clarifying the pathogenesis of PAAD and elucidating the molecular markers that are closely associated with disease progression are important ways to improve clinical PAAD treatment outcomes.

The critical role of the TME in oncogenesis, including its contribution to cancer progression and drug resistance [11–13]. Our study builds upon this foundation by focusing specifically on the interplay between autophagy and the TME in PAAD. Autophagy is a catabolic process by which cells can degrade proteins, organelles, and other biomolecules within the lysosome to promote cellular homeostasis [14]. Thus, cells perform quality control, and thus balance cell death and survival, primarily through autophagy, and the degraded and recycled metabolites can also provide energy for cell growth [15]. However, autophagy is a double‐edged sword that exhibits dual roles in different types of cancer. In the TME, autophagy can both increase the stress tolerance of tumor cells and maintain their survival in the tumor immune microenvironment, as well as suppress their generation and metastasis at different stages of tumor progression, and even serve as a death pathway for some apoptosis‐deficient tumor cells [16]. Moreover, in PAAD, autophagy is usually regarded as a prosurvival mechanism, which can help cancer cells to survive in the harsh microenvironment [17].

MicroRNAs (miRNAs) exert an imperative regulatory role in cancer cell autophagy and cancer progression [18, 19]. miRNAs are powerful genetic regulators, and individual miRNAs can direct entire cellular pathways through interacting with target genes, which endows miRNAs as an important tool for cancer therapy [20]. Studies have demonstrated that miRNAs function crucially in a variety of biological processes [21, 22]. In addition to the direct regulation of cancer progression, the regulatory features of miRNAs on cellular autophagy may also be explored for the development of clinical therapeutic strategies, given that miRNAs are key regulators of autophagy and are involved in multiple steps of autophagy [23]. Indeed, recent studies have demonstrated that targeting core autophagy‐related genes using miRNA‐based approaches can effectively suppress autophagy initiation or block autophagic flux in pancreatic cancer cells, leading to increased sensitivity to radiotherapy, chemotherapy, and other targeted agents [24]. Several specific miRNAs have been identified as critical modulators of autophagy in pancreatic cancer. For instance, miR‐138‐5p suppresses autophagy by targeting SIRT1, thereby inhibiting the SIRT1/FoxO1/Rab7 axis in pancreatic cancer cells [25]. Similarly, miR‐124 has been shown to restrain autophagy and suppress pancreatic cancer cell invasion and migration by directly targeting BECN1, a key autophagy‐related gene [26]. Additionally, mTOR, a central regulator of autophagy and also acts as an activator of the malignant phenotype of human pancreatic cancer cells, has been identified to be a target of various autophagy‐related miRNAs, including miR‐145, miR‐375, and miR‐3473b [27–30]. Taken together, the existing reports may be able to tap potential therapeutic targets for PAAD based on the characterization of miRNA regulation of cellular autophagy.

In this research, based on the weighted gene co‐expression network construction for The Cancer Genome Atlas (TCGA)‐PAAD, we elucidated the autophagy‐related characterized genes in PAAD samples. Moreover, these genes were markedly enriched for miRNA‐associated pathways and revealed four characterized genes as biomarkers associated with PAAD autophagy and progression. Further miRNA network construction elucidated the key miRNAs that have regulatory relationships with these biomarkers. Finally, molecular docking analysis based on the four biomarkers elucidated potential therapeutic drugs for PAAD. The molecular markers and drugs screened might contribute to the uncovering of the molecular mechanism of PAAD progression and the amelioration of clinical treatment strategies.

## 2. Materials and Methods

### 2.1. Data Acquisition

Expression data, including raw counts and fragments per kilobase of transcript per million mapped reads (FPKM), for PAAD were obtained from the Genomic Data Commons (GDC) Data Portal (https://portal.gdc.cancer.gov/). Only primary tumor samples corresponding to TCGA sample type code 01A were retained, yielding a total of 178 samples for subsequent analysis. Normal pancreatic tissue expression data were obtained from the Genotype‐Tissue Expression (GTEx) database (https://www.gtexportal.org/home%5B33), comprising 167 samples. The miRNA dataset GSE109319 was sourced from the Gene Expression Omnibus (GEO) database to obtain 24 pancreatic cancer tissue samples and 21 control samples. Finally, 367 autophagy‐related genes were acquired from the available literature [31].

### 2.2. Gene Sources

Using 367 autophagy‐related genes as the background gene set, single‐sample gene set enrichment analysis (ssGSEA) [32] was conducted by R software to assess the enrichment score for each sample in the background gene set. A total of 69 cancer stem cell (CSC) genes related to PAAD were obtained from published research [22, 33, 34].

### 2.3. Weighted Gene Co‐Expression Network Analysis (WGCNA)

The soft threshold (*β*) was determined for TCGA‐PAAD using the pickSoftThreshold function of the WGCNA package [35], and the intercept height was set to 0.9. Next, hierarchical clustering was used to find gene modules by setting the criterion of minModuleSize = 200, and the module merge height was set to 0.2. Subsequently, the modules were analyzed for correlation between the modules and autophagy correlation scores. We then selected the module most strongly correlated with the trait as the target module, calculated the gene significance (GS) and module membership (MM) values for genes in this module, and identified genes with |GS| ≥ 0.4 and |MM| ≥ 0.8 as hub genes.

### 2.4. Differential Expression Analysis and Key Gene Screening

The differentially expressed genes (DEGs) of PAAD samples compared to the control sample dataset were calculated by the R package DESeq2, and |log_2_FC| ≥ 1 and Padj < 0.01 were used as the screening criteria [36]. To further screen genes associated with cellular autophagy and PAAD progression, hub genes were intersected with DEGs to screen key genes.

### 2.5. Enrichment Analysis

In this study, the screened key genes were analyzed by Gene Ontology (GO) and Kyoto Encyclopedia of Genes and Genomes (KEGG) through the KOBAS‐i database (http://bioinfo.org/kobas), and the entries with *p* < 0.05 were retained. Pathway genes related to miRNA regulation and autophagy were selected as biomarkers.

### 2.6. Analysis of Immune Infiltration

Immune infiltration profile analysis was performed applying the CIBERSORT package [37] for R language software, and the correlation between biomarkers and immune cell infiltration profile was calculated based on Spearman’s correlation coefficient [3].

### 2.7. Regulatory Network Construction of Biomarkers and miRNAs

In this study, miRNAs targeting biomarkers were predicted by the Encori database (https://rnasysu.com/encori/), and the prediction results were imported into Cytoscape 3.8.0 to map the regulatory network of transcription factors, and labeled miRNAs that were significantly differentially expressed in the miRNA dataset and had an opposite expression trend to that of the targeting miRNAs that were significantly differentially expressed in the miRNA dataset and had opposite expression trends to the targeted biomarkers.

### 2.8. Targeted Drug Prediction and Molecular Docking

In this study, the R package Enrichr and Drug Signatures Database (DSigDB) were employed to analyze enriched gene sets and predict targeted drugs. Through the UniProt database, the crystal structures of receptor proteins were obtained. The 3D structures of the targeted drugs were downloaded as ligands from the PubChem website, according to the drug prediction results. All molecular structures were prepared using PyMOL [38] for dehydration, hydrogenation, and small‐molecule removal. The target drug’s 3D structure was then energy‐minimized with ChemBioOffice. Subsequently, receptor–ligand docking and screening were performed in AutoDockTools, applying the criteria of binding energy < −5 kcal/mol and hydrogen bond length ≤3.5 Å.

### 2.9. Cell Culture and Transfection

Human PAAD cell lines Capan‐2 (HTB‐80) and PANC‐1 (CRL‐1469) were purchased from ATCC (https://www.atcc.org/cell-products), and normal pancreatic duct epithelial cells HPDE6‐C7 (IM‐H374) were purchased from “immocell” (http://www.immocell.com/ryxbx/1384.html). Cells were cultivated in Dulbecco’s Modified Eagle Medium (DMEM) with 10% fetal bovine serum and 1% penicillin‐streptomycin, and cultured at 37°C in a 5% CO_2_ environment. siRNA was transfected into PANC‐1 and Capan‐2 cells using Lipofectamine 2000 transfection reagent kit (11668027, Invitrogen, Carlsbad, CA, USA). During transfection, siRNA was diluted to 100 nM, and Lipofectamine 2000 was diluted to 3 μL/well to achieve optimal transfection efficiency. After transfection, cells were stored at 37°C for 48 h. The siRNA sequences applied in this study are as follows (si‐*ZEB2*#1: TGGCCAAAACTACAAACAAGACT; si‐*ZEB2*#2: AGGAAAAACGTGGTGAACTATGA).

### 2.10. QRT‐PCR

Total RNA extracted from PAAD cells was reverse‐transcribed into cDNA and analyzed by qRT‐PCR. RNA quality was verified by measuring the OD260/OD280 ratio using a NanoDrop spectrophotometer. Amplification was performed using SYBR Green on an ABI 7500 system (Thermo Fisher Scientific, USA). The primer sequences in this study are as follows: *HGF* (F: GAGAGTTGGGTTCTTACTGCACG; R: CTCATCTCCTCTTCCGTGGACA); *RECK* (F: AATCCTTGCCCTGCCAATGAGC; R: GCACCTGGATTAGTGTCCCTTG); *CYP1B1* (F: GCCACTATCACTGACATCTTCGG; R: CACGACCTGATCCAATTCTGCC); *ZEB2* (F: AATGCACAGAGTGTGGCAAGGC; R: CTGCTGATGTGCGAACTGTAGG); *GAPDH* (F: GTCTCCTCTGACTTCAACAGCG; R: ACCACCCTGTTGCTGTAGCCAA).

### 2.11. Cell Counting Kit‐8 (CCK‐8) Test

The collected cell suspension was added to a 96‐well plate with 5 × 10^3^ cells per well and 100 μL per well. The CCK‐8 reagent was then injected and incubated for 30 min. Subsequently, the absorbance of each cell culture system at 450 nm was detected under a microplate reader (Bio‐Rad).

### 2.12. Scratch Healing Assay

The PAAD cell lines were seeded into six‐well plates, and 2 mL of complete medium was added to each well. The cells were cultured at 37°C in a 5% CO_2_ incubator and a pipette tip was applied to create straight scratches on the cell monolayer in each well. Thereafter, the cells were rinsed twice with phosphate‐buffered saline (PBS) to remove detached cell debris. The fresh serum‐free medium was further added to the cells for incubation. The migration of PAAD cells was finally photographed under a microscope at 0 and 48 h post‐scratch. Scratch healing rate = (initial scratch width − current scratch width)/initial scratch width × 100%.

### 2.13. Transwell Analysis

The PAAD cells were seeded into the upper chamber of a Transwell insert (Corning, USA) in the presence of serum‐containing medium in the lower chamber. After 48‐h culture at 37°C, the noninvading cells were dislodged from the upper chamber, and 4% paraformaldehyde was used to fix the cells, followed by coloring with crystal violet (10 min). Invaded cells were quantified using an inverted microscope.

### 2.14. Statistical Tests

All the data were analyzed with R software and GraphPad Prism software. The Wilcoxon rank sum test was used to calculate the difference between the two groups of continuous variables, and unpaired t‐test and one‐way or two‐way analysis of variance were applied to compare the experimental data. The results with *p*  < 0.05 were deemed to be statistically significant.

## 3. Results

### 3.1. Mining Autophagy‐Related Hub Genes in PAAD Based on WGCNA

We first obtained the ssGSEA scores of autophagy‐associated genes and jointly analyzed them by WGCNA to screen 14 modules by correlation analysis of each module, among which, the black module had a significant positive correlation with autophagy‐related ssGSEA scores, while the turquoise module exhibited a significant negative correlation with autophagy‐related ssGSEA scores, and thus these two modules were used as target modules in this study (Figure 1A, B). In this study, 158 hub genes were screened in these two modules based on the screening conditions of GS ≥ |0.4| and |MM| ≥ 0.8 (Figure 1C, D).

Figure 1Identification of autophagy‐related genes in PAAD based on WGCNA. (A) Analysis of the scale‐free fit index and average connectivity across various soft‐threshold powers (*β*). (B) Correlation analysis of each characterization module with autophagy‐related gene scores, with red denoting positive correlation and blue denoting negative correlation. (C, D) GS‐MM plots of the black module (C) and the turquoise module (D), with the horizontal coordinates as MM (module membership), that is the correlation of each gene with the characterized genes; vertical coordinate is GS (gene significance), that is the correlation of genes and traits within the module.

**Figure:**
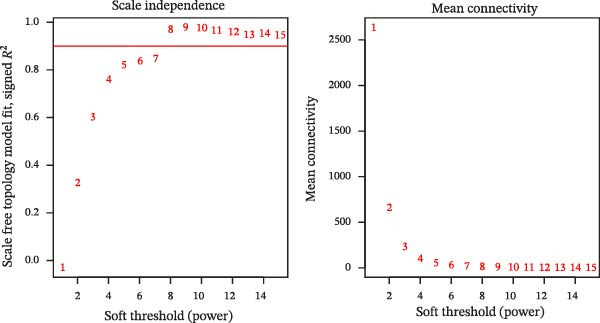
(A)

**Figure:**
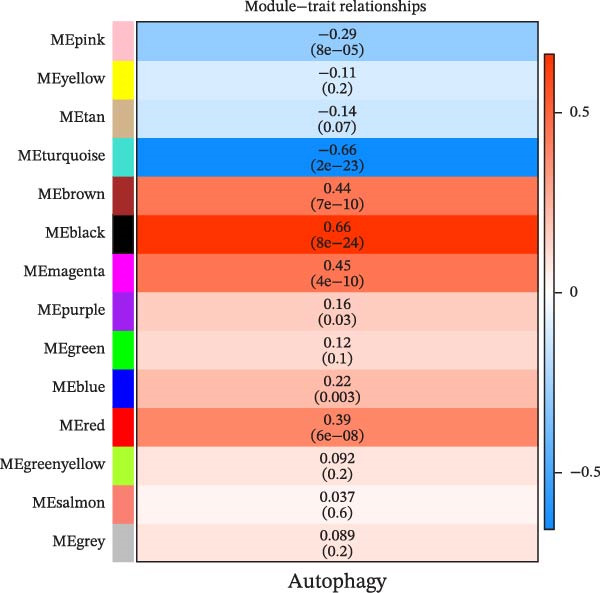
(B)

**Figure:**
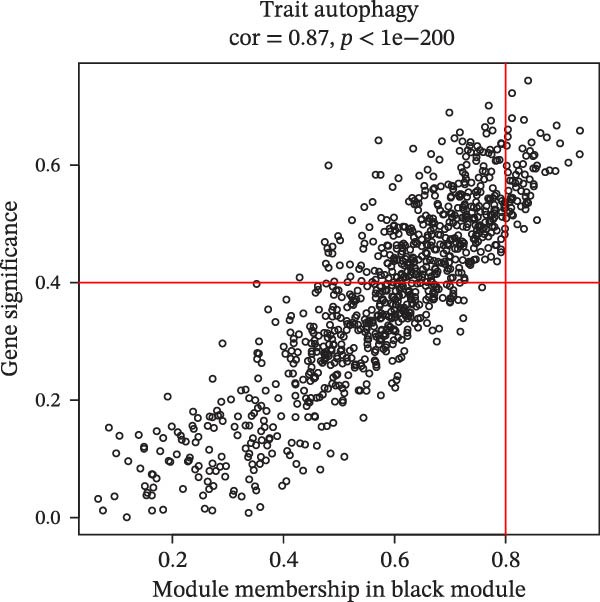
(C)

**Figure:**
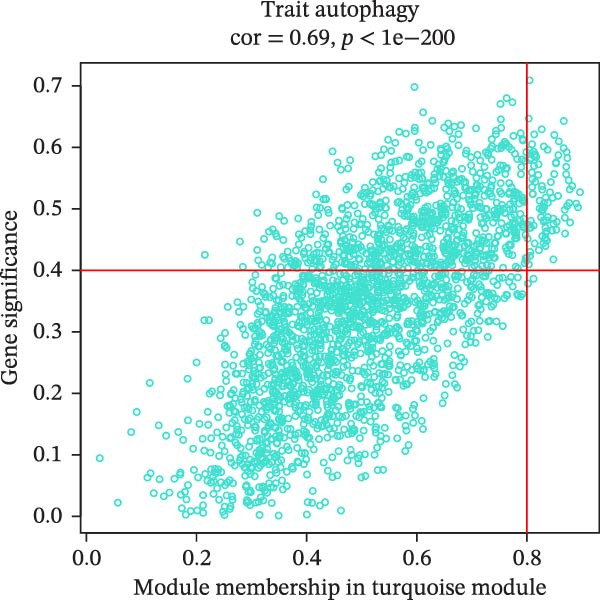
(D)

### 3.2. Screening of Key Genes Associated With Autophagy and PAAD Progression

A sum of 8340 DEGs, encompassing 3186 downregulated genes and 5154 upregulated genes, were filtered, and the gene differential expression volcano map is shown in Figure 2A. We further took the intersection of DEGs with the previously analyzed acquired hub genes, and finally obtained 61 key genes related to cellular autophagy and PAAD progression (Figure 2B). The expression heatmap of these genes was displayed in Figure 2C.

Figure 2Differential expression analysis. (A) Volcano plot of DEGs in PAAD compared to control samples, the color from blue to red represents the significance from low to high. (B) Intersection upset plot of differentially expressed genes in WGCNA’s hub genes with those in PAAD. (C) Differential expression heatmap of DEGs in PAAD intersecting with those in WGCNA’s hub genes.

**Figure:**
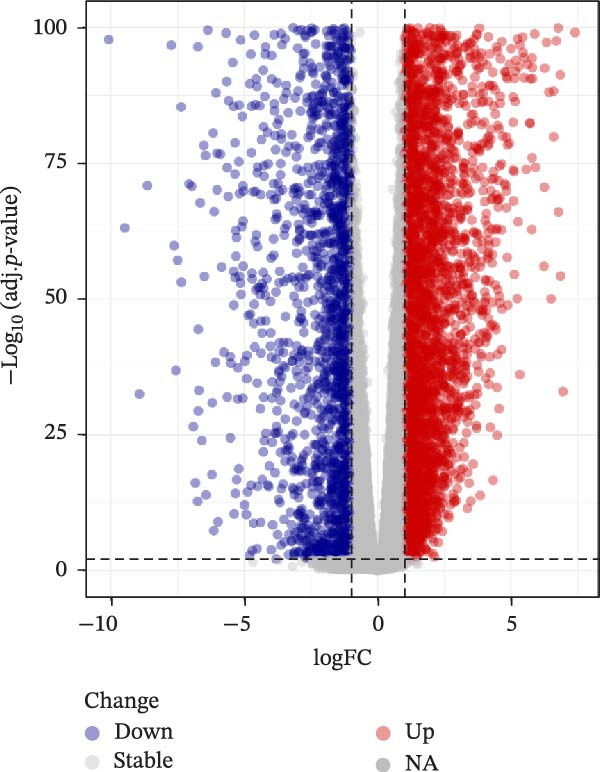
(A)

**Figure:**
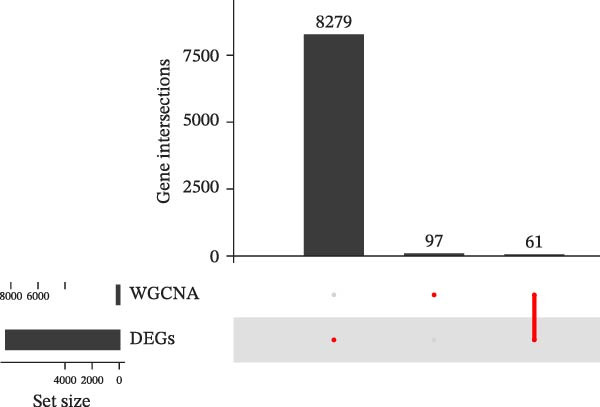
(B)

**Figure:**
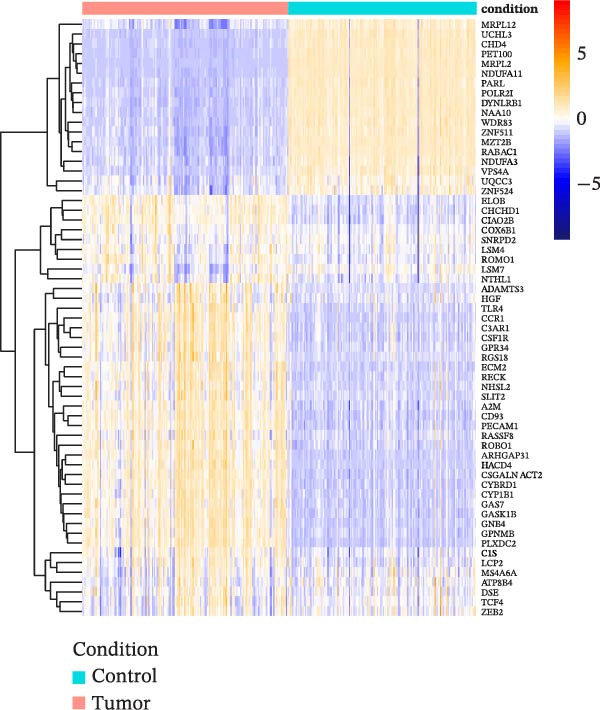
(C)

### 3.3. Functional Enrichment Analysis of Key Genes

In this study, enrichment analysis of 61 key genes associated with cellular autophagy and PAAD progression revealed that these genes were primarily implicated in the positive regulation of vascular endothelial growth factor signaling pathway, phagocytosis, and miRNAs in cancer pathway (Figure 3A–D). Among them, the miRNAs in cancer pathway contained four genes, *HGF*, *RECK*, *CYP1B1*, and *ZEB2*, which were proposed to be used as biomarkers for subsequent validation in this study in view of the close association of miRNAs with the progression of PAAD and the regulation of autophagy.

Figure 3Gene function enrichment analysis. (A) GO‐BP analysis of intersecting genes. (B) GO‐CC analysis of intersecting genes. (C) GO‐MF analysis of intersecting genes. (D) KEGG analysis of intersecting genes. Horizontal coordinates represent gene number in the entries, color indicates the significant *p* value, with increasing significance from blue to red.

**Figure:**
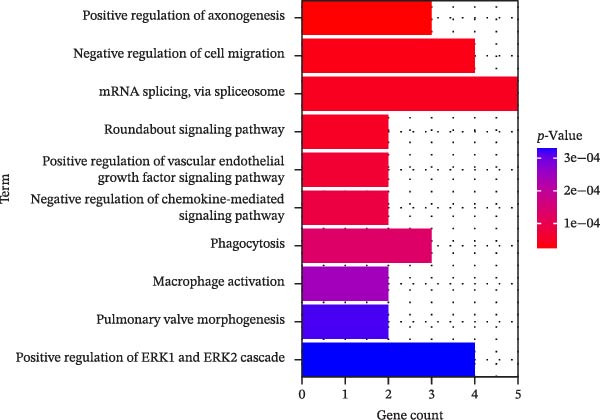
(A)

**Figure:**
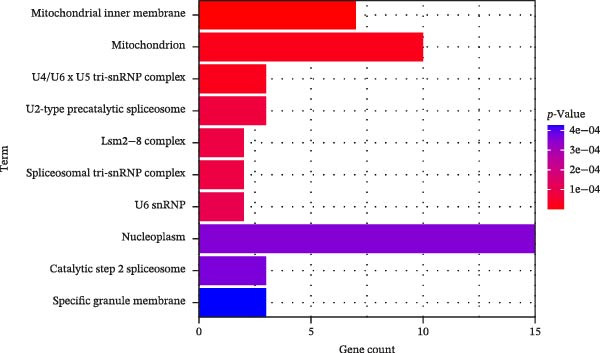
(B)

**Figure:**
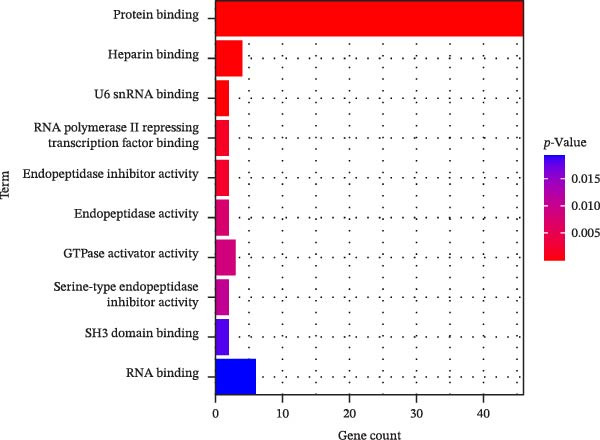
(C)

**Figure:**
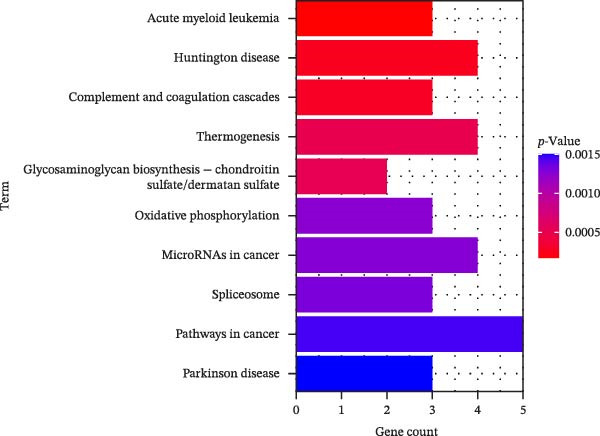
(D)

### 3.4. Correlation of Biomarkers Associated With Autophagy and miRNA Regulation in PAAD With Immune Infiltration

Immune infiltration analysis was conducted on TCGA samples employing CIBERSORT, and correlation coefficients between biomarker expression and immune infiltration scores were calculated. The results displayed that the expression levels of *HGF*, *RECK*, *CYP1B1*, and *ZEB2* were notably associated with the infiltration of most immune cells, for example, they were significantly positively linked to the infiltration scores of cells such as T cells CD4 memory activated and T cells CD8, while they were markedly negatively correlated with the infiltration scores of cells such as Tregs and NK cells activated (Figure 4A). ESTIMATE analysis displayed that the expressions of these biomarkers were all closely positively linked to the immune‐related indexes of PAAD (Stromal, Immune, ESTIMATE scores) (Figure 4B). This also implies that PAAD progression and cellular autophagy‐related biomarkers may have a potential regulatory role in immune cell infiltration.

Figure 4Correlation analysis of biomarkers and immune infiltration. (A) The Spearman correlation analysis of four biomarkers with CIBERSORT immune infiltration. (B) The Spearman correlation analysis of four biomarkers with ESTIMATE immune infiltration,  ^∗^ *p* < 0.05,  ^∗∗^ *p* < 0.01, and  ^∗∗∗^ *p* < 0.001.

**Figure:**
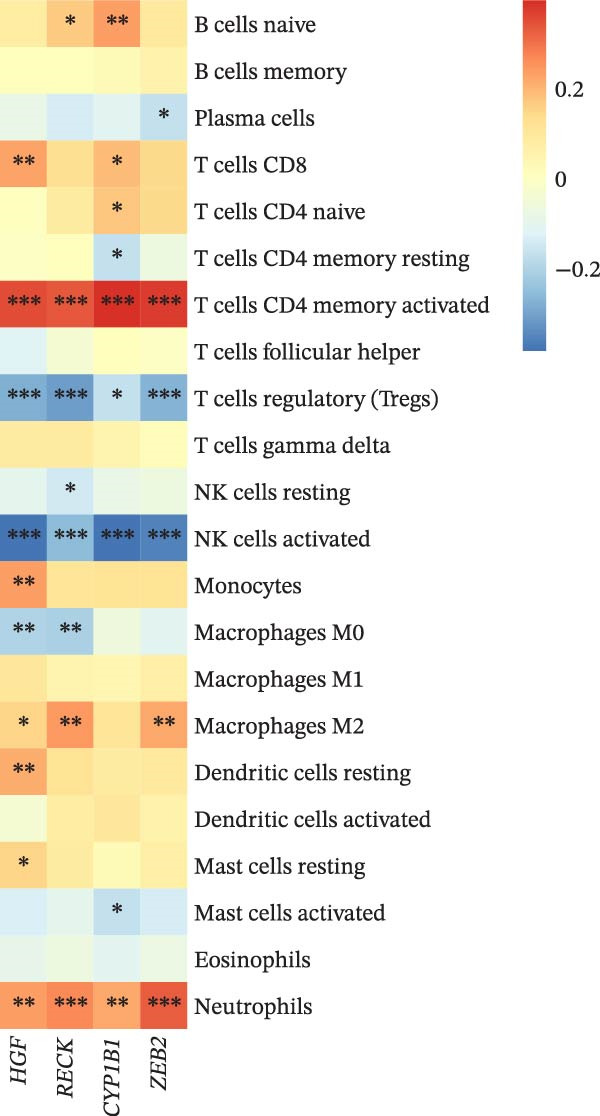
(A)

**Figure:**
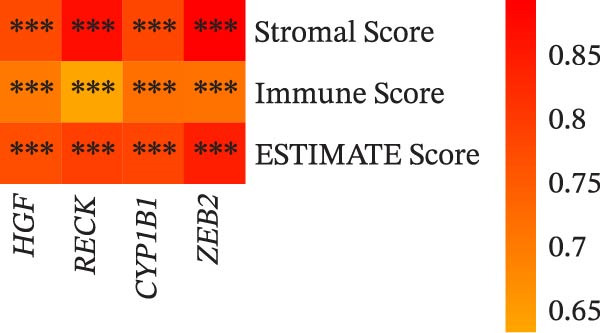
(B)

### 3.5. Regulatory Network Construction of Biomarkers and miRNAs in PAAD

**Figure 5:**
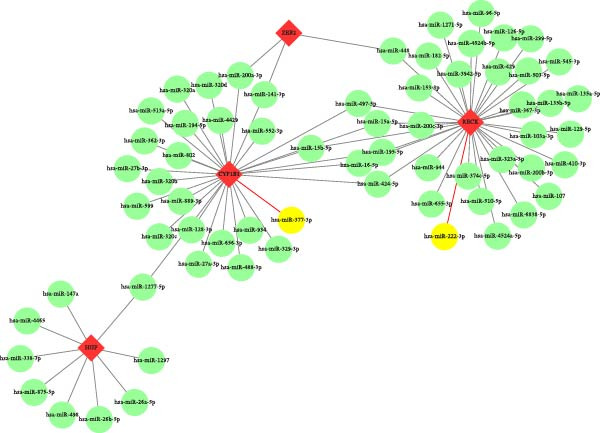
miRNAs regulatory network construction. Red diamonds represent biomarkers, green circles represent miRNAs, and yellow circles represent miRNAs that are differentially expressed and have an opposite expression trend to biomarkers.

In this study, a search of Encori database showed that there were 9 target miRNAs validated by 5 or more experiments for *HGF*, 35 for *RECK*, 29 for *CYP1B1*, and 3 for *ZEB2*. Further analysis of the miRNA dataset revealed, among others, that the relative expression level of hsa‐miR‐222‐3p in PAAD had an opposite expression trend to its target *RECK*, while the same relationship existed between hsa‐miR‐377‐3p and *CYP1B1*, validating the targeting relationship between these specific miRNAs and downstream biomarkers (Figure 5).

### 3.6. Drug Prediction and Molecular Docking for Targeting Biomarkers in PAAD

In this study, we predicted biomarkers for targeting drugs in DSigDB database and combined it with molecular docking for drug screening and prediction. The results of molecular docking demonstrated that *CYP1B1*, *HGF*, and *RECK* all have a stable binding relationship with the drug resveratrol (Figure 6A–C). Among them, the binding energy of *CYP1B1* to resveratrol was −6.86 kcal/mol, that of *HGF* to resveratrol was −6.9 kcal/mol, and that of *RECK* to resveratrol was −5.58 kcal/mol (Table 1). The remaining parameters are shown in Table 2. These outcomes also implied that resveratrol may exert a potential role in PAAD treatment.

Figure 6PAAD potential drug prediction and molecular docking. (A) Molecular docking of CYP1B1 with resveratrol. (B) Molecular docking of *HGF* with resveratrol. (C) Molecular docking of *RECK* and resveratrol, depicting the receptor (blue), ligand (green), key residues (lime green), and hydrogen bonds (yellow dashed lines with lengths in Å).

**Figure:**
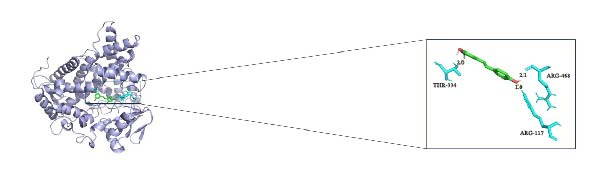
(A)

**Figure:**
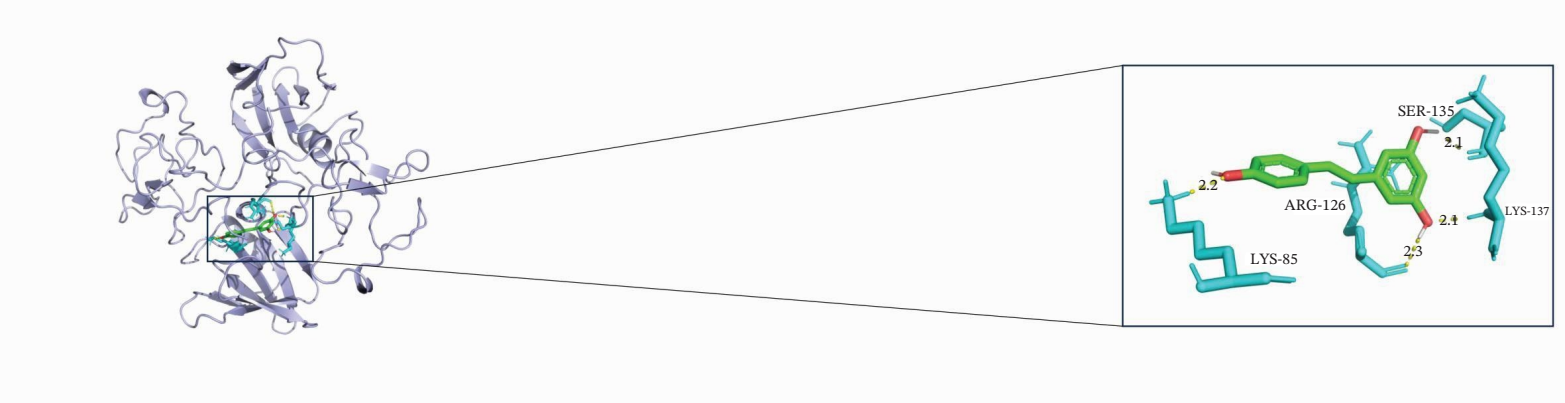
(B)

**Figure:**
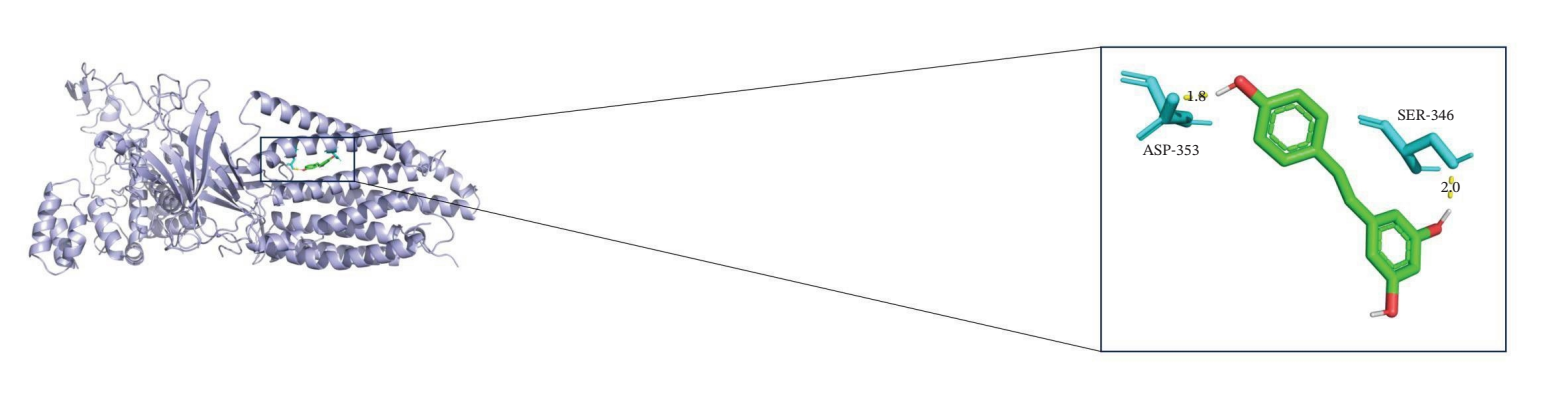
(C)

**Table 1:** Binding energies of the molecular docking.

Compound CID	Molecula_name	Gene_name	PDB_ID	Energy (kcal/mol)
445154	Resveratrol	*CYP1B1*	3pm0	−6.86
445154	Resveratrol	*HGF*	1bht	−6.9
445154	Resveratrol	*RECK*	8tzp	−5.58

**Table 2:** Parameters of the molecular docking.

Term	Spacing	npts	Center
CYP1B1_resveratrol	0.625	126, 126, 126	−21.671, 17.822, −13.148
HGF_resveratrol	0.525	126, 126, 126	67.781, 24.789, 58.531
RECK_resveratrol	1.000	126, 126, 126	129.035, 132.592, 141.778

### 3.7. Biomarkers Were Positively Correlated With CSC in PAAD

The CSC scores of 69 genes in TCGA‐PAAD samples were calculated through ssGSEA. Correlation analysis between four biomarkers (*CYP1B1*, *HGF*, *ZEB2*, and *RECK*) and CSC scores was conducted using the Spearman algorithm. The results showed that *HGF*, *RECK*, and *ZEB2* were significantly positively correlated with CSC scores (*R* > 0.3, Figure 7A–D. The results of GSEA enrichment analysis showed that the high expressions of *HGF*, *RECK*, and *ZEB2* genes all led to the significant enrichment of EPITHELIAL_MESENCHYMAL_TRANSITION and TGF_BETA_SIGNALING (Figure 7E–G). Among them, EPITHELIAL_MESENCHYMAL_TRANSITION is a key process for tumor stem cells to acquire migratory, invasive, and metastatic abilities, and directly participates in the characteristics of tumor stem cells.

Figure 7Biomarkers were positively correlated with CSC in PAAD. Scatter plot of the correlation between CYP1B1 (A), *HGF* (B), *RECK* (C), *ZEB2* (D) and the score of CSC. GSEA enrichment analysis of *HGF* (E), *RECK* (F), and *ZEB2* (G) genes.

**Figure:**
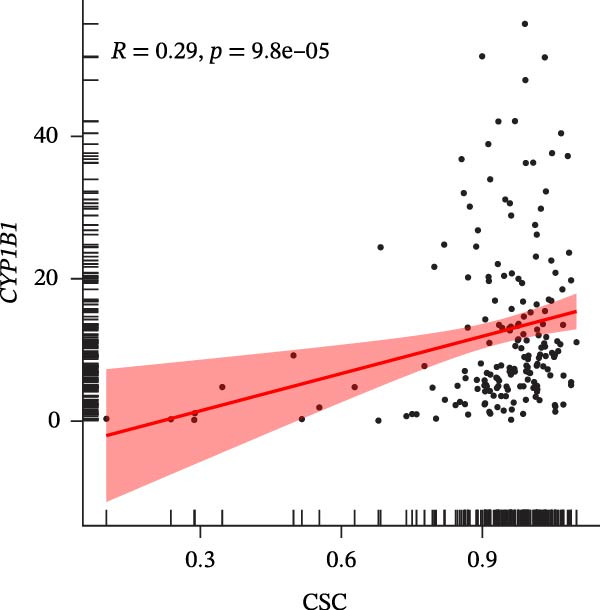
(A)

**Figure:**
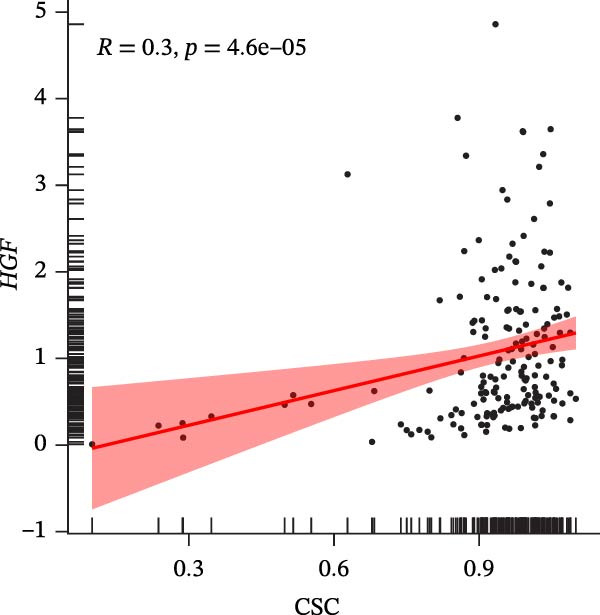
(B)

**Figure:**
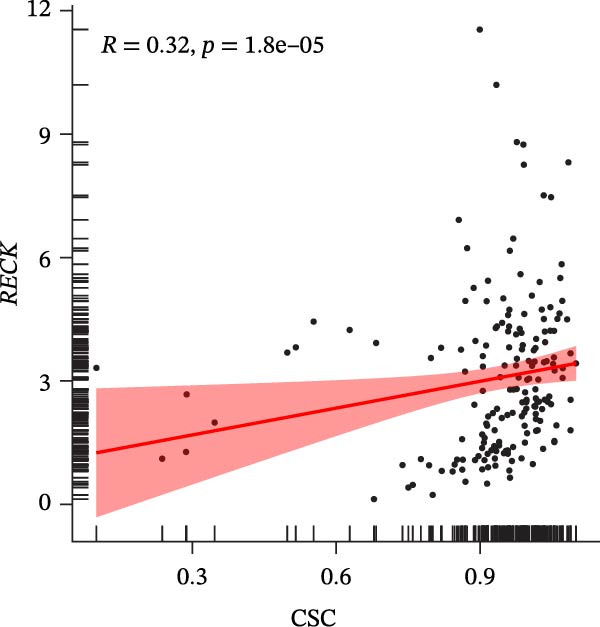
(C)

**Figure:**
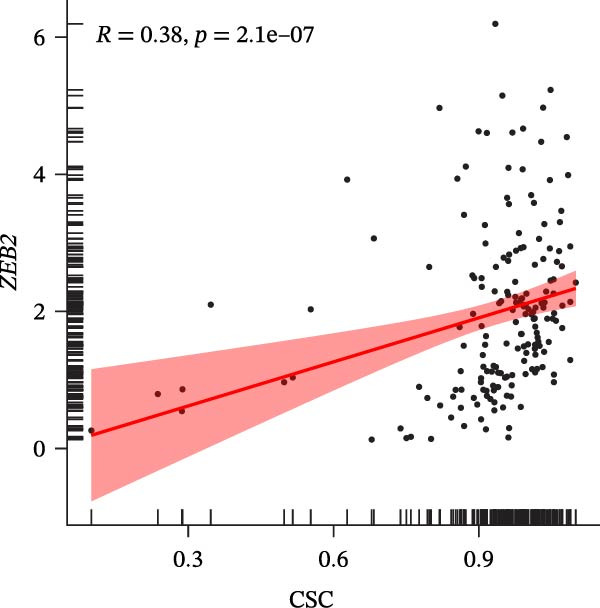
(D)

**Figure:**
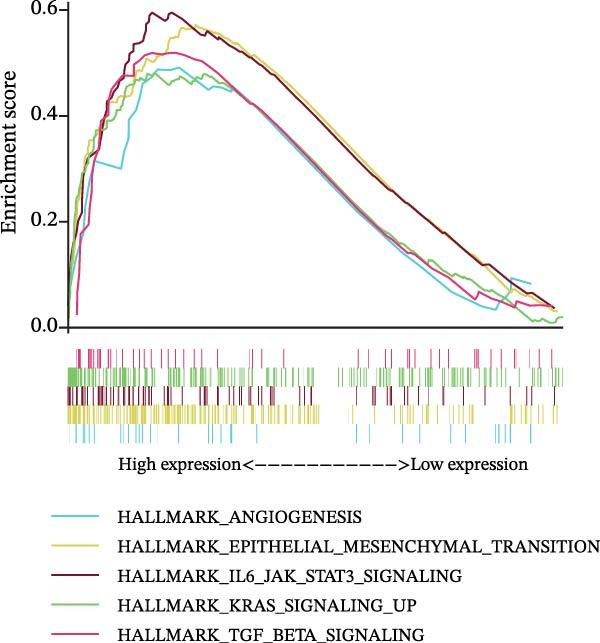
(E)

**Figure:**
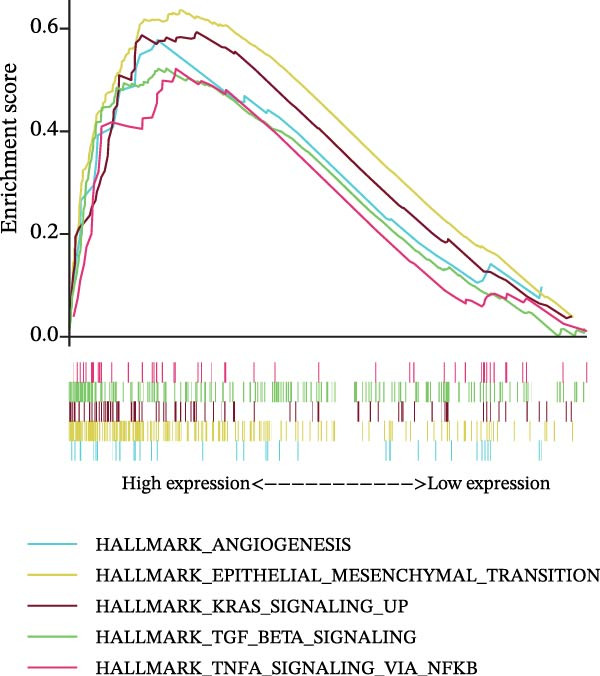
(F)

**Figure:**
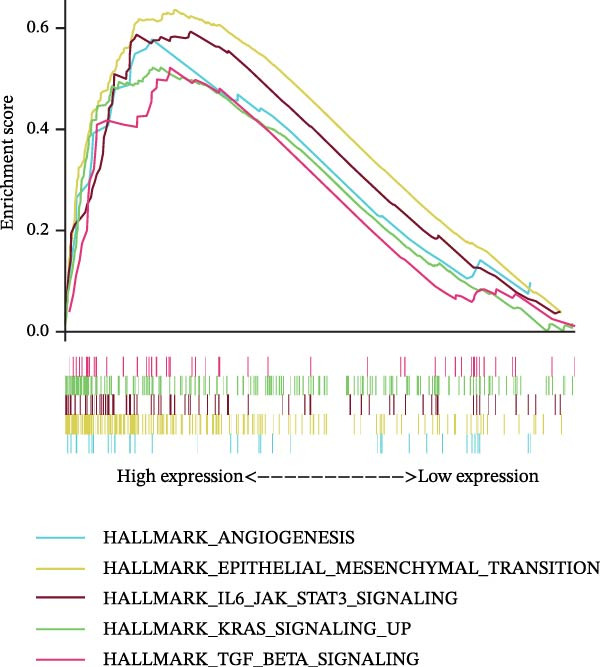
(G)

### 3.8. The Regulatory Role of PAAD Biomarkers in PAAD Cell Lines

This study used molecular detection to confirm that *ZEB2* is notably upregulated in PAAD cells (Figure 8A). Existing literature reports that *ZEB2*, as a key EMT transcription factor, plays a central role in tumor invasion and metastasis; however, current evidence regarding its regulatory functions in PAAD cells remains insufficient. Thus, PAAD cells PANC‐1/si‐*ZEB2* and Capan‐2/si‐*ZEB2* were constructed with *ZEB2* silenced (Figure 8B, C). CCK‐8 assays displayed that *ZEB2* silencing downregulated the proliferation of PAAD cell lines (Figure 8D, E). *ZEB2* silencing, in the meantime, also inhibited the migratory and invasive capabilities of PAAD cell lines, as evidenced by wound healing and Transwell assays (Figure 8F–I).

Figure 8Regulatory effect of the biomarkers on PAAD cells. (A) Relative expressions of four biomarkers in different PAAD cell lines. (B) Construction of the *ZEB2*‐silenced PAAD cell line PANC‐1/si‐*ZEB2*. (C) Construction of the *ZEB2*‐silenced PAAD cell line Capan‐2/si‐*ZEB2*. (D) CCK‐8 test to assess the relative proliferation levels of PANC‐1/si‐*ZEB2*. (E) CCK‐8 test to assess the relative proliferation levels of Capan‐2/si‐*ZEB2*. (F) Wound healing test to determine the migration levels of PANC‐1/si‐*ZEB2*. (G) Transwell experiment to measure the invasion level of PANC‐1/si‐*ZEB2*. (H) Wound healing test to measure the migration level of Capan‐2/si‐*ZEB2*. (I) Transwell experiment to measure the invasion level of Capan‐2/si‐*ZEB2*. ns: no significance,  ^∗∗∗∗^ *p*  < 0.0001,  ^∗∗∗^ *p*  < 0.001,  ^∗∗^ *p*  < 0.01,  ^∗^ *p*  < 0.05.

**Figure:**
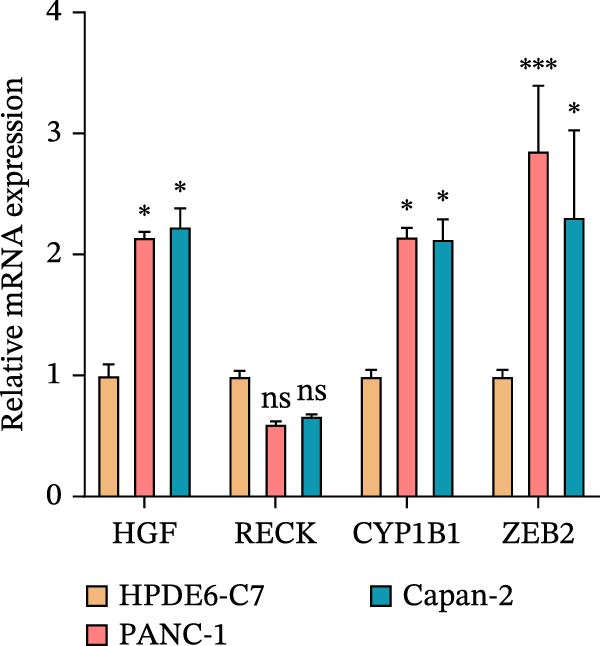
(A)

**Figure:**
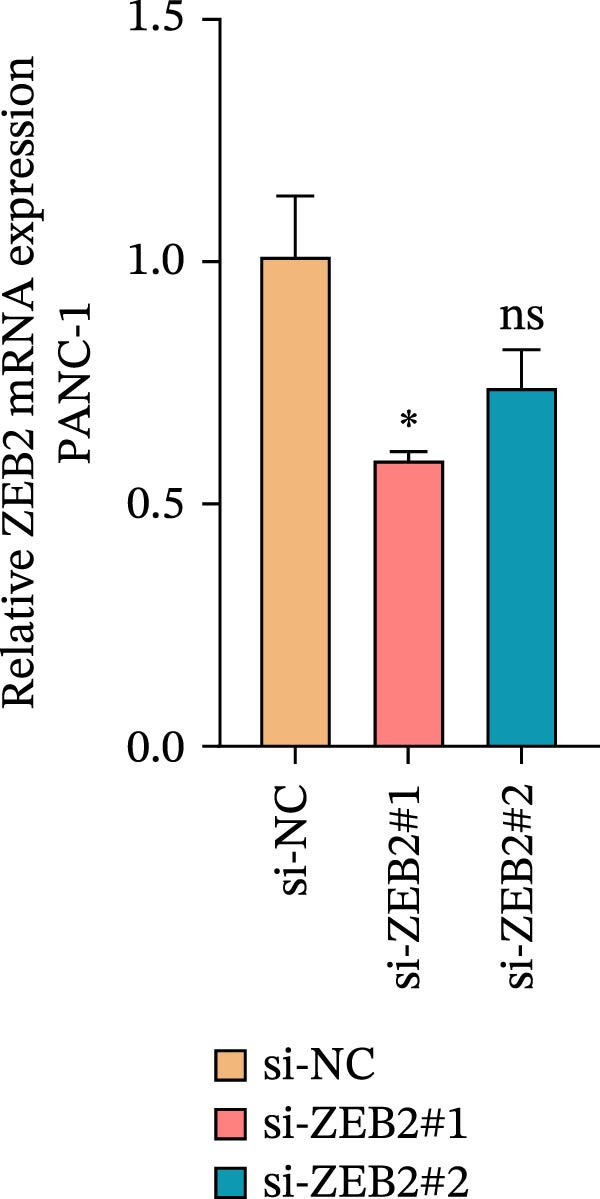
(B)

**Figure:**
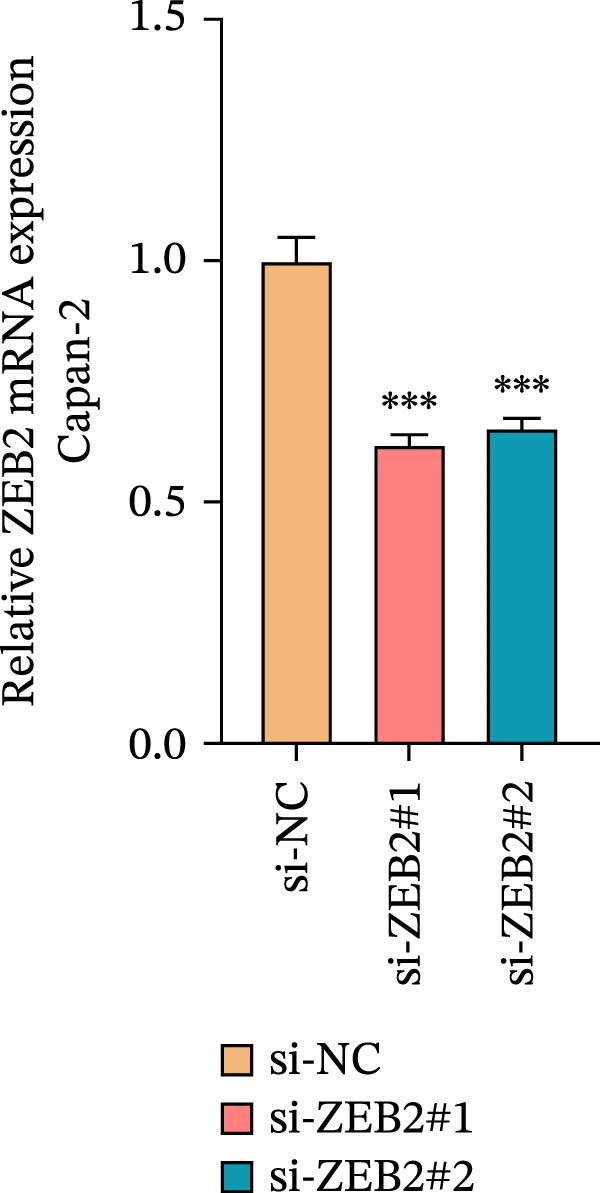
(C)

**Figure:**
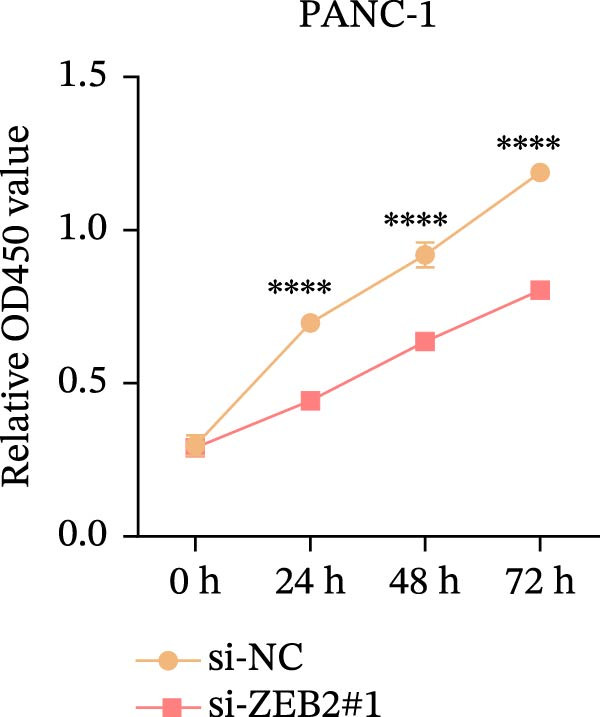
(D)

**Figure:**
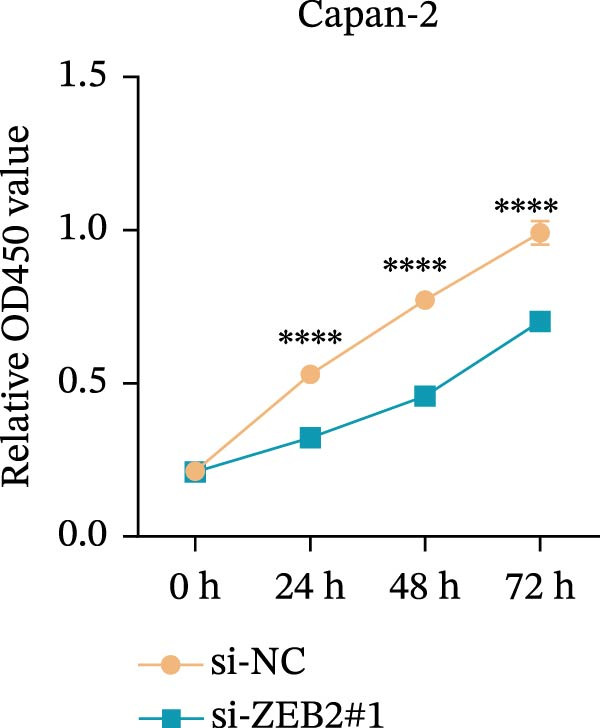
(E)

**Figure:**
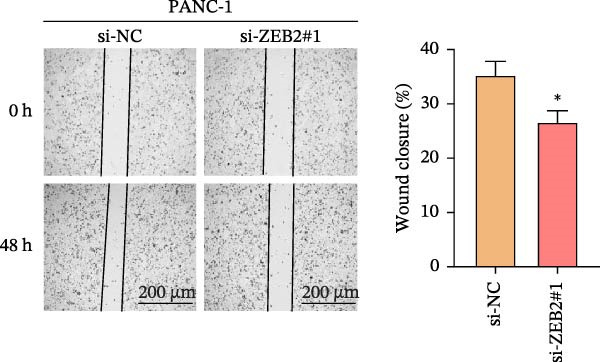
(F)

**Figure:**
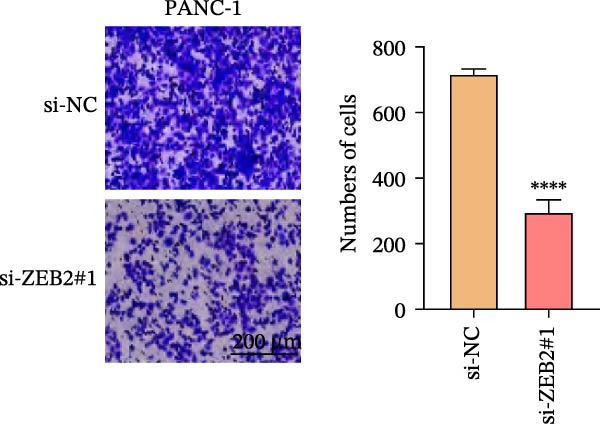
(G)

**Figure:**
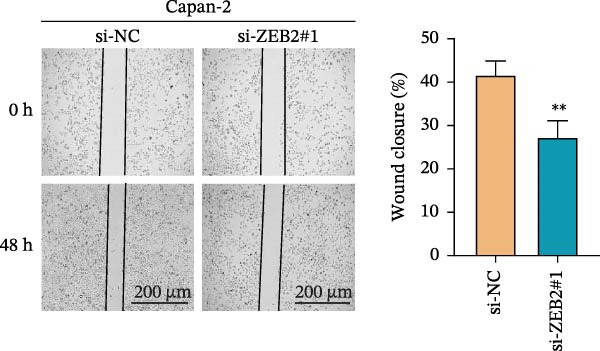
(H)

**Figure:**
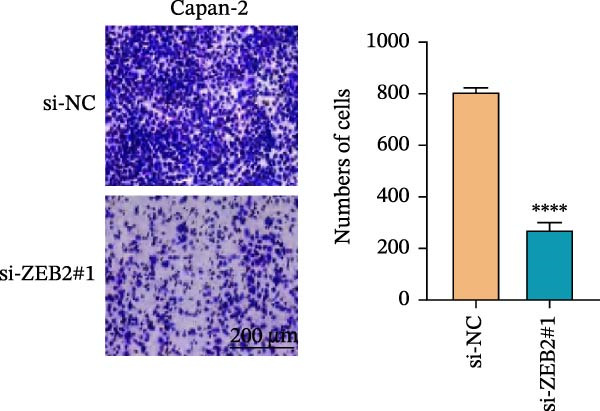
(I)

## 4. Discussion

PAAD, as one of the deadliest tumors, has long lacked effective therapeutic means, and the lack of effective clinical targets makes it difficult to carry out targeted treatment. Relevant reports have found that cellular autophagy‐related features are of great significance in revealing the mechanism of PAAD development. For example, cellular autophagy usually involves the formation of autophagy‐specific VPS34 complexes, including components of VPS34, beclin‐1, ATG14, etc., and the expression of these proteins correlates with cancer cell proliferation, migration, apoptosis, and chemo‐resistant phenotypes, as well as involves the activation of downstream cancer‐related pathways, which is expected to be a promising treatment target for cancers [39–42]. Here, this study focuses on autophagy‐related genes in PAAD, employing a combined approach of WGCNA and differential expression analysis to identify target module genes. It reveals that these module‐specific genes are significantly enriched in the miRNAs in cancer pathway, thereby identifying key regulatory genes within this pathway as potential biomarkers for subsequent research. Building upon this foundation, the study not only elucidates the regulatory relationships between these biomarkers and miRNAs but also identifies potential targeted therapeutics through molecular docking techniques. This approach provides novel insights for exploring PAAD treatment strategies from dual perspectives—autophagy regulation and miRNA networks—while laying a theoretical groundwork for enhancing PAAD clinical efficacy.

In this study, four biomarkers (*HGF*, *RECK*, *CYP1B1*, and *ZEB2*) associated with autophagy characterization and miRNA regulation in PAAD were mined. Among them, the hepatocyte growth factor *HGF* is a pro‐oncogene in PAAD that regulates cell growth, motility, and morphogenesis in multiple cancer cells and tissue types [43]. This factor has a regulatory relationship with the epithelial transforming factor c‐MET, and a related study demonstrated the antitumor activity of HGF/MET pathway inhibitors in PAAD [44]. The activation of the HGF/MET pathway can mediate cells to enter the autophagy‐dependent degradation pathway [45]. *RECK* is downregulated and expressed in numerous human cancers, and in PAAD, a related study demonstrated by animal experiments that pancreatic *RECK* deletion significantly increased the development of PAAD with a mesenchymal phenotype, which was accompanied by an increase in liver metastasis from PAAD and a decreased survival rate in mice decreased [46]. Regarding the regulatory mechanism, downregulated expression of *RECK* was associated with higher expression levels of matrix metalloproteins such as MMP2 and MMP3, which are proteases involved in extracellular matrix degradation and basement membrane disruption that regulate cellular autophagy and modulate malignant tumor cell invasion and metastasis [47]. The upstream of *RECK* is regulated by miRNAs such as miR‐21, which is linked to cancer cell growth and metastasis [48]. The *CYP1B1* is upregulated and expressed in various cancers, like subkidney, breast, and colorectal cancers, and is directly relevant to the poor prognosis of patients [49–51]. Downstream of this molecule, it induces activation of the mTOR pathway, which in turn can block cellular autophagy [52, 53]. *ZEB2* is a DNA‐binding transcription factor primarily implicated in EMT, whereas *ZEB2* acts as a key role in EMT‐induced cell differentiation and malignant phenotypes [54]. The upstream regulatory miRNAs of *ZEB2* include miRNA‐1179, miRNA‐155, and miRNA‐206 [55–57]. Among them, miRNA‐155 regulates cellular autophagy and thus affects cellular oxidative damage [58]. Through the existing reports, it can be seen that the biomarkers explored in this study are all associated with cancer progression and cellular autophagy regulation, or there is a regulatory relationship with miRNAs, which is consistent with the findings of this study.

In this study, we uncovered the binding relationship between hsa‐miR‐222‐3p and *RECK* in PAAD and the targeting relationship between hsa‐miR‐377‐3p and *CYP1B1* through the construction of a network of miRNAs. hsa‐miR‐222‐3p is a potential prognostic factor for cancers such as hepatocellular carcinoma and lung cancer [59]. Overexpression of clustered hsa‐miR‐221 and hsa‐miR‐222 has been demonstrated to directly result in the downregulation of tumor suppressor and cell cycle regulator p27 [60]. Downstream of hsa‐miR‐377‐3 p can target oncogene E2F3 to regulate cancer cell growth in vitro and tumor growth and metastasis in vivo [61]. In addition, this study predicted by molecular docking that *CYP1B1*, *HGF*, and *RECK* all have stable binding relationships with the drug resveratrol. Resveratrol is expected to ameliorate cancer and facilitate breakthroughs in cancer therapy by acting as a chemical prophylactic agent in the four major stages of cancer (initiation, promotion, progression, and metastasis) and by showing both in vitro and in vivo efficacy in cancer therapy [62]. With its antioxidant, anti‐inflammatory, and antitumor properties, this drug has great potential as a complementary agent to conventional chemotherapy [63].

However, there are some limitations in this study. First, the data for this study primarily originate from public databases, presenting limitations in sample size and platform heterogeneity. Future research should collect large‐scale tissue samples through multicenter clinical cohorts for external validation. Additionally, techniques such as immunohistochemistry should be employed to validate the expression of the four characteristic genes and their clinical relevance in independent cohorts. Second, the construction of the miRNA regulatory network relies on target gene prediction databases and lacks experimental validation. Subsequent studies should verify the direct targeting relationships between hsa‐miR‐222‐3p and *RECK*, as well as hsa‐miR‐377‐3p and *CYP1B1*, using methods such as luciferase reporter assays in PAAD cell models. Third, molecular docking predictions only indicate potential binding between resveratrol and target proteins. However, its actual antitumor efficacy and mechanism of action in PAAD remain experimentally unconfirmed. Subsequent studies should evaluate its therapeutic effects through cell viability assays, apoptosis analysis, and in vivo tumorigenesis experiments in nude mice. Finally, the immune infiltration analysis is based on transcriptomic data and lacks in situ evidence of the tumor immune microenvironment. Subsequent validation at the tissue level is required to confirm the correlation between marker expression and immune cell infiltration using techniques such as multiplex immunofluorescence or flow cytometry.

## 5. Conclusion

Collectively, we identified four autophagy‐related signature genes (*HGF*, *RECK*, *CYP1B1*, and *ZEB2*) in PAAD through public databases and bioinformatics analysis. These genes are significantly enriched in the miRNAs in cancer pathway and are positively correlated with immune infiltration and CSC scores in PAAD. A miRNA regulatory network was constructed, revealing targeting relationships between hsa‐miR‐222‐3p and *RECK*, as well as hsa‐miR‐377‐3p and *CYP1B1*. Molecular docking analysis predicted that resveratrol binds stably to *CYP1B1*, *HGF*, and *RECK*. In vitro experiments demonstrated that silencing *ZEB2* significantly inhibits the proliferation, migration, and invasion of PAAD cells. These findings provide new insights into autophagy‐related mechanisms in PAAD and offer a theoretical basis for developing targeted therapies and prognostic biomarkers.

**Nomenclature**
GEO: Gene Expression Omnibus
GO: Gene Ontology
GS: Gene significance
GTEx: Genetype‐Tissue Expression
KEGG: Kyoto Encyclopedia of Genes and Genomes
MM: Module membership
miRNA: MicroRNA
PAAD: Pancreatic adenocarcinoma
ssGSEA: Single‐sample gene set enrichment analysis
TCGA: The Cancer Genome Atlas
WGCNA: Weighted gene co‐expression network analysis.

## Author Contributions

Zhige Yu and Hongxi Chen designed the study. Shenglei Song and Hongxi Chen acquired the data. Hongxi Chen interpreted the data. Hongxi Chen, Shenglei Song, and Chenglong Li drafted the manuscript. Hongxi Chen, Chenglong Li, and Zhige Yu revised the manuscript.

## Funding

The study was supported by grants from the Scientific Research Project of the Health Commission of Hunan Province (Grant B202304016521), the doctoral fund and the 2020 Cultivation Project for the National Natural Science Foundation of China from the People’s Hospital of Hunan Province (Grant BSJJ202012) and Hunan Provincial Natural Science Foundation Project (Grant 2026JJ82667) with Hongxi Chen.

## Disclosure

All authors read and approved the manuscript.

## Ethics Statement

Ethical approval was not required for this study because it did not involve any human experiments.

## Consent

The authors have nothing to report.

## Conflicts of Interest

The authors declare no conflicts of interest.

## Data Availability

The datasets generated and/or analyzed during the current study are available in the (GSE109319) repository (https://www.ncbi.nlm.nih.gov/geo/query/acc.cgi?acc= GSE109319).
